# Experiences With Health Care Services in Switzerland Among Immigrant Women With Chronic Illnesses

**DOI:** 10.3389/fpubh.2020.553438

**Published:** 2020-10-16

**Authors:** Annika Frahsa, Romaine Farquet, Tevfik Bayram, Luna De Araujo, Sophie Meyer, Sibel Sakarya, Sandro Cattacin, Thomas Abel

**Affiliations:** ^1^Institute of Sport Science, University of Tübingen, Tübingen, Germany; ^2^Institute of Social and Preventive Medicine, University of Bern, Bern, Switzerland; ^3^School of Medicine, Department of Public Health, Marmara University, Istanbul, Turkey; ^4^Institut de Recherches Sociologiques, Université de Genève, Geneva, Switzerland; ^5^School of Medicine, Department of Public Health, Koç University, Istanbul, Turkey

**Keywords:** chronic care, women, immigrant backgrounds, quality of care, access to care, policy transfer, participatory planning, PREs

## Abstract

**Introduction:** Descriptive data indicate a high burden of chronic illness among immigrant women in Switzerland. Little is known about how immigrant women with chronic illnesses experience healthcare services. This paper presents a methodological approach theoretically informed by Sen's capability approach and Levesque's framework of access to healthcare to study patient-reported experiences (PREs) of Swiss healthcare services among immigrant women with chronic conditions.

**Methods:** We conducted 48 semi-structured qualitative interviews in Bern and Geneva with Turkish (*n* = 12), Portuguese (*n* = 12), German (*n* = 12), and Swiss (*n* = 12) women. Participants were heterogenous in age, length of stay, SES, and educational attainment, illness types and history. We also conducted semi-structured interviews with healthcare and social service providers (*n* = 12). Interviewed women participated in two focus group discussions (*n* = 15). Interviews were transcribed verbatim and analyzed using Atlas.ti software, based on Gale et al.'s framework approach. Findings informed three stakeholder dialogues in which women as well as healthcare providers and policymakers from various territorial levels participated.

**Results:** Our methodological approach succeeded in integrating women's perspectives—from initial data collection in interviews to identify issues, focus group discussions to increase rigor, and stakeholder dialogues to develop tailored recommendations based on PREs.

**Discussion:** This is one of the first studies in Switzerland that used PREs to research healthcare services and healthcare needs among immigrant women with chronic illnesses. This paper provides new insights on how to better understand existing challenges and potentially improve access to and quality of care.

## Introduction

Switzerland is one of the most expensive, but also one of the highest developed healthcare systems. It is a combination of public, subsidized private, and fully private elements. One need for coordination stems from the federalist organization of the Swiss health system that has divided responsibilities between the federal, the 26 cantonal, and the local levels. Concretely, the Swiss healthcare system is characterized by a compulsory basic health insurance scheme, with similar insurance premiums for all, but also subsidies for the poorer inhabitants, and are accompanied by further patient payments in forms of an annual excess (the deductible) and a charge of 10% of all basic costs that exceed the excess (excluded are some expensive treatments and hospital interventions). The compulsory basic insurance can be supplemented by private complementary health insurance, for which more than 20 insurance companies offer diverse schemes. The complementary insurance is to cover additional treatment dimensions and to improve standards of rooms and services in case of hospitalization. These basic insurance finances around 50% of the costs of the health system. The other 50% is financed by income-based taxes (in particular for hospital infrastructures, civil servants in the health system, and public health services).

However, even if each person, independent of his or her legal status, can access basic health services, access remains limited for some population groups and healthcare needs (1). In Switzerland and other European countries, health inequities have been attributed to legal status, socioeconomic status (SES), migration-related risk factors, low health competence, and health system factors (2–7). Although Switzerland's migrant population is heterogeneous and includes migrants with higher SES, some migrants from Turkey, Portugal, the former Yugoslavia, Sri Lanka, and different Sub-Saharan African countries are more disadvantaged and more likely to have worse health conditions than the average Swiss citizen (8). Moreover, immigrants without a stay permission (sans-papiers) do not tend to purchase the health insurances that are expensive, and for this group, non-subsidized, but rather depend on private and public *ad hoc* health services, most notably offered in urban areas. Descriptive data indicate low health literacy and potential over- and underuse of services among some migrant subpopulations (3, 9, 10). Furthermore, the burden of disease is especially high among migrant women, and certain immigrant groups are more likely to have chronic conditions (8).

Sen's seminal capability approach links structural conditions within the healthcare system to the particular experiences of people who aim to access or use healthcare services (11–13). We use the capability approach as a theoretical framework to understand the tension that shapes users' real opportunities. Likewise, in using the capability approach, we also focus on the interplay between the utilization of services and healthcare service infrastructure. In healthcare research, care services are often operationalized using Levesque's framework of access to healthcare (14). To understand the links between healthcare services, utilization and equity, research must also consider patients' experience of illness, especially for chronic conditions (15, 16). Studying illness from immigrant patients' perspectives can reveal the complex social, economic, and cultural conditions and processes associated with the experience of immigration (17–19). We also need to understand how patients' and providers' perspectives on health services can be linked to identify and suggest solutions for complex problems in healthcare systems (20–22).

Haslbeck et al. (23) showed that the knowledge base on chronic illness experience is constantly growing. Studies have explored patients' access to adequate support within a mostly fragmented health system. Studies have focused on challenges in finding relevant health information and on collaboration with healthcare providers. The monitoring of symptoms, management of medication regimens, as well as dealing with uncertainty and other difficult emotions, and integration of disease-related tasks into everyday life has also been explored (23). However, so far, very few studies have explicitly addressed a “patient view” among immigrant populations or the structural challenges immigrants living with chronic illness face in Switzerland.

For the purpose of this study, we adopted a broad definition of the term “chronic illness” referring to non-communicable health problems lasting for more than a year and diagnosed by a physician in Switzerland or abroad and self-reported by the patient.

This paper aims to address the gap in health care research on how to integrate patients' perspective on structural challenges in access to and quality of care for immigrants living with chronic illness. Therefore, this paper will present the specific theory-informed methodological approach of a study that aims to understand the experiences of women with chronic illness and immigration background and to use selected results to highlight the potential benefits and challenges linked to such an approach. The paper will firstly present study design, setting, and methods for data collection and analysis, used in the study. Secondly, this paper will present selected results on the recruitment of women with chronic illness and immigration experience as well as on the integration data from different qualitative data sources to identify practical implications of system barriers, as well as individual and social resources available to make best use of the services provided. The paper will close by a discussion of benefits and challenges of this approach used in the study.

## Methods

### Study Design

**Figure 1:**
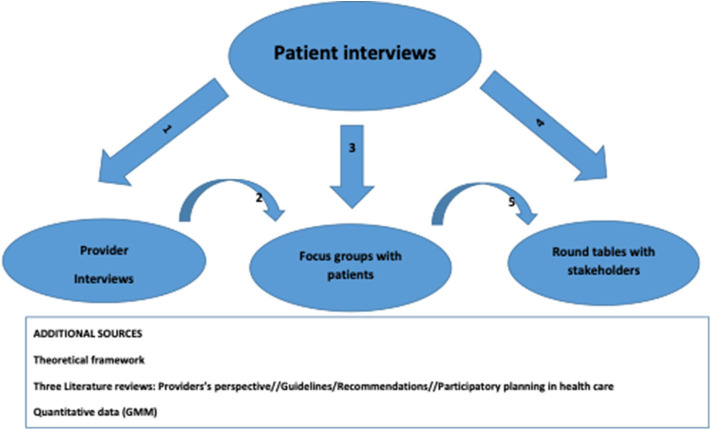
Data flows from different sources in MIWOCA.

The methodological approach and selected results presented in this paper stem from the MIWOCA study. MIWOCA is short for “Migrant women's healthcare needs for chronic illness services in Switzerland.” MIWOCA is a multi-method qualitative study in two cantons of Switzerland (Bern and Geneva). The study aims to improve the understanding of the healthcare service experiences of immigrant women with chronic illnesses living in Switzerland. MIWOCA uses Sen's seminal capability approach as theoretical framework and builds upon Levesque's model of access to healthcare to research patient-reported experiences (PREs) of Swiss healthcare services among immigrant women with chronic conditions. PREs are used in health research to capture patient perspectives on various aspects of care and aid in evaluating the overall quality of health services (24, 25). The MIWOCA study is a multi-method qualitative study. It integrates different data: semi-structured guideline-based qualitative interviews with women with chronic illnesses and with healthcare and social service providers, focus group discussions with women interviewed, as well as stakeholder dialogues, to develop a set of practice recommendations. Figure 1 shows the different data flows. We used the data and knowledge gained from the previous data source to elaborate and reflect on the data as well as to broaden the knowledge base in MIWOCA.

We conducted 48 interviews with chronically ill women to capture the perspectives of women with chronic conditions. Secondly, we conducted twelve interviews with healthcare and social services providers to explore providers' experiences in providing health and social services to chronically ill patients. Interviews with providers had two specific objectives: (1) to capture their vision of systemic factors facilitating or hindering access to healthcare for chronically ill persons, with a particular focus on potential system improvements (26) and (2) to compare and contrast the women's viewpoints with the providers' perspectives. Thus, the interviews also sought to capture the providers' opinion on specific issues previously mentioned by the women. Thirdly, we organized two focus group discussions (in Bern and Geneva) to present our results to the women who had previously participated in the interviews. The women had the opportunity to discuss the results and express their views on potential issues that should be further discussed in stakeholder dialogues. Lastly, we conducted stakeholder dialogues where professionals in healthcare and social services, as well as women who participated in interviews, met. These dialogues were conducted over the course of three meetings and participants developed shared recommendations for better access to and better quality of healthcare for people with chronic conditions.

### Study Setting

MIWOCA took place in the Swiss cantons of Bern and Geneva to understand how chronically ill women manage their illness in two different societal and healthcare contexts (27).

#### Sampling, Recruitment and Conducting of Interviews With Women

MIWOCA focused on two different population groups: (1) women with an immigration background and (2) women without such background, who had been born in Switzerland and lived there for most of their life. Within the first group, we included women from three distinct immigrant sub-populations: first-generation Portuguese, Turkish, and German immigrants. These three countries are among the most common countries of origin of immigrants living in Switzerland [Germany, second most common (14.7%); Portugal, third most common (12.7%), Turkey, seventh most common (3.2%)] (28). Immigrants from these countries show similarities, but also differences in terms of health, healthcare utilization, and potential cultural and social determinants (3, 8–10).

We also included native Swiss women in our study to differentiate generalizable conditions of chronic disease patients (i.e., burdens, barriers, resources and strategies) from conditions unique to those with an immigration background.

We followed a purposive, a priori-defined maximum-variation sampling strategy. We aimed to include women who had immigrated to Switzerland from Turkey (*n* = 12), Portugal (*n* = 12), and Germany (*n* = 12). The inclusion criteria for this sampling-group were as follows: (1) first-generation immigrants who had entered Switzerland after compulsory schooling, (2) aged 18 years and older, and (3) with at least one medically diagnosed chronic disease (e.g., migraine, diabetes, depression, or chronic pain). Cancer was excluded because it leaves limited decision-making leeway to patients.

In addition to the first group of participants, Swiss women (*n* = 12) with similar chronic diseases were also included in the study. These were Swiss nationals who had been born in Switzerland and had spent most of their lives in this country. In addition, the objective was to select a heterogeneous sample in terms of age, length of stay if with an immigration background, type and length of illness, SES and educational attainment. We pre-defined 48 interviews (12 for each group) as a saturation point on the basis of previously conducted qualitative studies (29).

Other studies on topics similar to MIWOCA indicated that meaningful results were reached by similar sample sizes (30, 31). We used different recruitment strategies to reach interviewees: personal contacts via researchers' professional and private networks, cultural associations, labor unions, associations for the elderly and retirement homes, academic institutes, hospitals, physiotherapists, and physicians or specialists known to have many immigrant patients and/or command of those patients' native languages. We also recruited via public leaflets in shops, restaurants, pharmacies, churches etc., social media advertisements, and through snowballing by interviewees. All participants received a small gift box upon completion of the interview.

**Figure 2:**
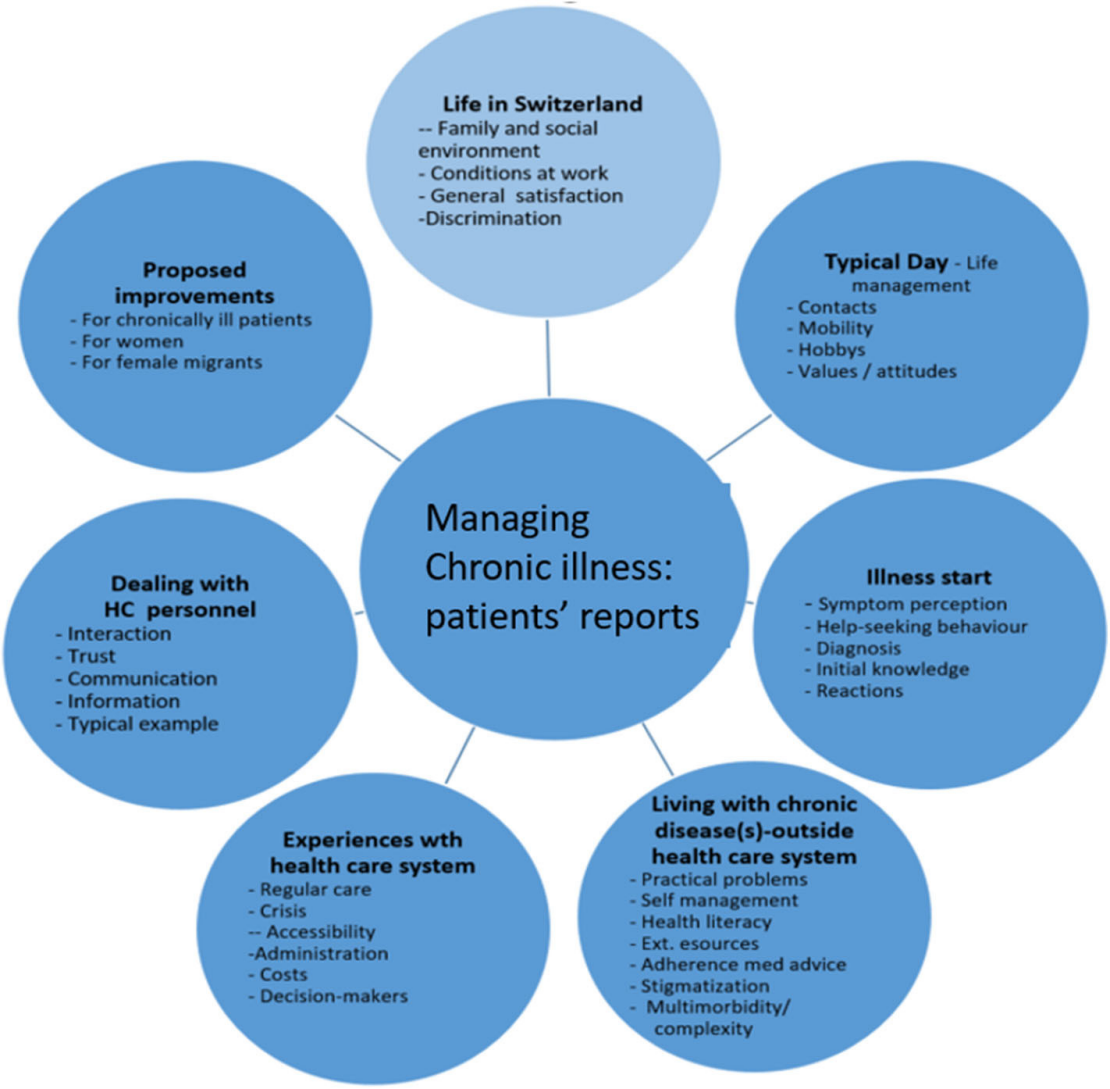
Main topics and themes of the interview guidelines.

Based on the existing literature and findings from surveys, the research team developed a semi-structured interview guideline to facilitate theme-oriented interviews. Figure 2 presents the seven main topics and respective themes included in the interview guide. We constructed the topics and themes to meet the dimensions and domains of Sen's approach and Levesque's framework (11–14). Questions asked referred to their perception of living in Switzerland, managing daily life, how their illness began, what living with a chronic condition is like, their experiences with the healthcare system, interaction with doctors and healthcare personnel, potentially typical experience with the healthcare system, and main messages regarding system improvements (for detailed information on interview questions see supplementary file 1, the interview guidelines for interviews with women).

We also included a standardized short questionnaire on the socio-demographics of the participants in the interview guideline.

Four researchers based at Swiss and Turkish universities conducted the interviews according to the different language requirements. A researcher (woman, professor in public health, MD) native in Turkish language conducted the interviews with Turkish women in Turkish in both Bern and Geneva. Another researcher (woman, background in anthropology and sociology) native in French and Portuguese conducted interviews with Swiss women in French language in Geneva as well as with women in Portuguese language in both Bern and Geneva. A third female researcher (background in sociology) conducted interviews with women in German language in both Bern and Geneva. Finally, a fourth interviewer (woman, background in sociology and migration studies) fluent in German and Swiss German was responsible for the three interviewees with women in a Swiss German dialect in Bern. The four interviewers sought to guarantee similar interview practices by communicating regularly with each other and exchanging problems and questions related to issues covered. Likewise, they maintained consistency when formulating questions and establishing relationships with research participants. The research participants were free to choose the place and time of the interview. Interviews were conducted largely at their homes, some at their workplace, others at our university offices, and a few in cafés. On a few occasions, the interviewees were accompanied by members of their family or acquaintances. On average, the formal and recorded part of the interviews lasted 75 min.

#### Sampling, Recruitment and Conducting Interviews With Healthcare Providers

The number of professionals interviewed (*n* = 12) had been pre-defined prior to the start of the study; the sampling criteria were mainly determined based on results from the interviews with women. Interview coders screened their interviews, listed the different types of providers mentioned, and refined those lists through team members' own insights and experiences, as well as available survey data on population health in Switzerland (8, 32). Through this process, researchers selected a purposive sample to include the perspectives of general practitioners, specialists, therapists, and social workers. Providers were recruited via personal and professional networks in Bern and Geneva. In a few cases, providers who were interviewed had already been contacted in the recruiting phase of the migrant women.

The interview guideline for providers was based upon several sources, including: MIWOCA team expertise, reports by women interviewed, the Levesque model, a literature review on providers' perspectives, and recommendations by MIWOCA advisory board members. Questions in the interviews referred to their experience in providing care to chronically ill patients, their perception of system barriers and facilitators that impact the opportunities of women (born abroad and with chronic illness) to use healthcare services, needs, challenges, and difficulties of these patient groups, and their suggestions for future system improvements (cf. supplementary file 2, interview guideline for service providers). Two researchers, separately based at Geneva and Bern universities, conducted the interviews. The interviews were conducted at the providers' offices. On average, the formal and recorded part of the interviews lasted 1 hour.

#### Focus Groups Discussions

We invited all interviewed women who had agreed to be contacted again to participate in focus group discussions (FGDs). Of the women interviewed in Bern, 17 expressed interest in participating, and of those interviewed in Geneva, 15 expressed interest. Participants were contacted via email, postal letters, phone calls, or messenger services. As some participants were more comfortable communicating in their native language, the respective MIWOCA interviewer contacted them in their native language to inform them about the FGDs. A total of ten women participated in the FGD in Bern and a total of five women participated in the FGD in Geneva. FGDs were conducted in German and French, respectively, in accordance with the official language of the two Swiss cantons and to allow discussion between women with different countries of origin. The FGD in Bern was held at the Institute of Social and Preventive Medicine at the University of Bern. All participants were paid 100 SF and 10SF for transportation expenses upon completion of the FGD.

Each FDG was conducted by one of the authors, with a second author as co-conductor. All of the co-authors are experienced in conducting interviews and focus groups. As noted above, each FGD was conducted in the language most comfortable for the greatest number of participants. In addition, a translator was present during the FGDs upon request by the participants. For this reason, a Turkish-German translator participated in the FGD in Bern.

The FGDs considered health services issues identified in the interviews that called for improvements. We presented three stimuli to the participants related to (1) access to healthcare, (2) interactions with healthcare providers, and (3) solutions to deal with issues highlighted in the initial interviews with the women. Each stimulus was accompanied by a list of bullet points providing more differentiated elements of that specific issue.

FGDs lasted 120 min, including a short break. During the discussions, we audio-recorded and had a written record, took field notes, and produced thematic maps to visualize issues the women addressed. At the end of the FGDs, participants visually prioritized issues by assigning points to them. We prepared and sent out a summary of the FGDs to participants to get feedback and ensure that we considered the points relevant to them.

#### Stakeholder Dialogues

MIWOCA stakeholder dialogues were conducted as part of a participatory planning process. Relevant stakeholders, including experts from health services and interviewed women, discussed the study results with researchers. These dialogues were used to triangulate findings, identify key stakeholders and priority areas for action, and jointly develop a concrete set of recommendations for improving Swiss healthcare services to respond more readily to women's needs for chronic illness healthcare.

We used purposive sampling to include patients in the stakeholder dialogues. All five FGD participants in Geneva expressed interest in the stakeholder dialogues and three FGD participants in Bern volunteered to represent the women's voice. We then considered diversity in countries of origin and types of illnesses for the final invitations. We also invited representatives from relevant institutions at the national and canton levels, both governmental and non-governmental. Additionally, to ensure a mixture of levels and sectors involved, we invited representatives from selected health insurance companies and regional/local associations in healthcare and social services (a detailed list of participants is available upon request).

Three half-day long stakeholder dialogues took place at the Institute of Social and Preventive Medicine at the University of Bern between September 2019 and January 2020. A person could chose to attend one, two, or all three stakeholder dialogues. Each dialogue was dedicated to one specific priority issue identified in the interviews and focus groups. The number of participants for each dialogue varied between 20 (first meeting), 13 (second meeting), and 25 (third meeting). The stakeholder dialogues were facilitated by the first author, and co-facilitated by the PI and co-PI of MIWOCA.

Prior to the stakeholder dialogue, participants received the MIWOCA brief. The brief summarized findings from the study, presented narrative stories of women's experiences, and highlighted main issues to improve access to and quality of healthcare services. Each stakeholder dialogue focussed on one of three main issues identified. The first dialogue focused on patients' needs, including their own competences and comprehension/shared understanding of the service system. The second dialogue focused on existing resources and strategies and how to optimize healthcare processes to improve their quality. The final dialogue presented a preliminary set of recommendations based on the study and dialogue findings, which were then discussed, adapted, and agreed upon as relevant and transferrable. In addition, we collected ideas and suggestions for dissemination and implementation of the recommendations from the group of participants.

### Data Analysis

We analyzed the interview data using Atlas.ti software and based on the seven steps of the framework method (33): transcription, familiarization, coding and categorizing, developing a working analytical framework, applying the framework, data charting, data interpretation. We transcribed interviews verbatim and translated transcripts into English. Three coders did deductive and inductive coding of three sample interviews (one Turkish, one Portuguese, one German). Dimensions of the Levesque model of access to healthcare served as a deductive set of categories, whereas content not described in the Levesque model was used in inductive coding. Subsequently, some of the pre-coded interviews were re-coded by a second researcher and the reliability between the two coders was checked. Given the generally high agreement between coders, remaining interviews were coded by single researchers. Our analytic process was iterative, with the primary objective of identifying themes, relationships, and patterns in the accounts of immigrant women with chronic conditions and in the data we gathered from interviews with health/social service providers.

## Selected First Results

In the field of patient-centered research, MIWOCA added a focus on women's practical experience with chronic illness and migration, as well as their subjectively perceived barriers and resources to healthcare. MIWOCA integrated data from different qualitative data sources to identify practical implications of system barriers, as well as individual and social resources available to make best use of the services provided. In the following, we will present selected first results: (1) on the recruitment of women with chronic illness and immigration experience, (2) the need to consider strategies and resources used by patients in dealing with healthcare services, and (3) the content of the practice recommendations developed during the stakeholder dialogues and further steps.

### Successful Recruitment

MIWOCA was successful in recruiting women across language groups and location of residence in Switzerland. Table 1 shows that participating women were very heterogenous in terms of age, length of stay, socio-economic status, educational attainment, as well as types of illnesses and illness history. The youngest participant was 23 years old and the oldest was 86 years old. In terms of length of stay, the participants had been living in Switzerland between 8 months and 60 years. While some participants lived on social welfare, others held full time managerial positions. Similarly, educational attainment varied between compulsory school to PhD levels. In terms of illnesses, most interviewees presented a relatively long disease history of several years and were multimorbid. As such, they were usually quite experienced in living with chronic illnesses. Some of the most frequent illnesses mentioned were: chronic pain, depression, asthma, hypertension, and migraine.

**Table 1:** Characteristics of women interviewed.

		**Turkish**	**Portuguese**	**German**	**Swiss**	**Total**
Number of interviews		12	12	12	12	48
Mean age	In years	53.2	52.3	52.7	55.8	53.5
Range	In years	41–71	46–62	28–86	23–85	23–86
Educational states	Low	8	6	0	1	15
	Middle	2	4	3	5	14
	High	2	2	9	6	19
Chronic conditions	One	1	3	3	5	12
	Two	1	6	5	2	14
	Three or more	10	3	4	5	22
Type of condition	Chronic pain (incl. Back pain)	6	5	3	2	16
	Depression	4	2	0	3	9
	Asthma/Shortness of breath	2	1	2	3	8
	Hypertension	5	0	2	1	8
	Migraines	1	3	1	2	7
	Diabetes (type I + II)	1	1	2	1	5
	Thyroid diseases	3	0	2	0	5
	Ostheoarthritis	0	3	0	2	5
	Fibromyalgia	2	2	0	1	5
	Allergies	0	1	1	2	4
	Anxiety/tension/panic attack	2	1	0	1	4
	Arthritis/Athrosis/polyarthritis	2		1	1	4
	CVD (carotid, bypass, lymphedema)	2	1	1	0	4
	Herniated disc/hip dysplasia/spine/Shoulder	2		2	0	4
	Crohn's disease/Ulcerative colitis	0	1		2	3
	Discal hernia	2	1	0	0	3
	Foot issues (paresis, hallux, Morton syndrome)	0	0	2	1	3
	Hypercholesterolemia	3	0	0	0	3
	Rheumatism	3	0	0	0	3
	Anemia/iron deficiency anemia	2	0	0	0	2
	Hypothyroidism	2	0	0	0	2
	Lichen sclerosis/psoriasis	0	0	1	1	2
	Multiple sclerosis	0	0	0	2	2
	Autoimmune disease	0	0	1	0	1
	Bipolar disorder	0	0	1	0	1
	Carpus	0	0	1	0	1
	Chronic sinusitis	0	1	0	0	1
	Convulsion	0	0	1	0	1
	Coughing	0	0	1	0	1
	Digestive problems	0	0	0	1	1
	Dizziness	0	0	1	0	1
	Endometriosis	0	0	0	1	1
	Face neuralgia	0	0	1	0	1
	Gallbladder	1	0	0	0	1
	Hiatal hernia	0	1	0	0	1
	Idiopathic pulmonary fibrosis	1	0	0	0	1
	Knee problems	0	0	1	0	1
	Liver problems	0	1	0	0	1
	MI/bypass	1	0	0	0	1
	Osteoporosis	0	0	1	0	1
	Portuguese amyloid neurpathy	0	1	0	0	1
	Premenstrual dysphoric disorder	0	0	1	0	1
	Psychiatric disease	1	0	0	0	1
	Tinnitus	0	1	0	0	1
	Underweight	0	0	0	1	1
	Urinary incontinance	1	0	0	0	1
	Total mentions	49	27	30	28	134

While recruiting participants from Turkey and Switzerland was fairly easy, recruiting women from Germany and Portugal was more challenging. This was largely due to the fact that German women often did not categorize themselves as “immigrants,” a term they tended to link to refugees. They also preferred to go unnoticed within the Swiss German-speaking population and not to be addressed as Germans. On the other hand, Portuguese women with often multiple (sometimes undeclared) employments or care tasks tended to lack resources, trust, or interest to participate in interviews.

For the interviews with healthcare and social service providers, we succeeded to interview twelve healthcare and social services providers, male and female, as planned. We interviewed two general practitioners, one gynecologist, one rheumatologist/allergist, one neurologist, and one home care nurse in Geneva. We interviewed one general practitioner (Turkish speaking), one social worker, one psychologist, one orthopaedist, one psychotherapist, and one physiotherapist/alternative medicine provider in Bern. Several providers who were interviewed had immigrated parents or were themselves immigrants.

### Revisiting Strategies and Resources Used by Patients in Dealing With Healthcare Services

**Figure 3:**
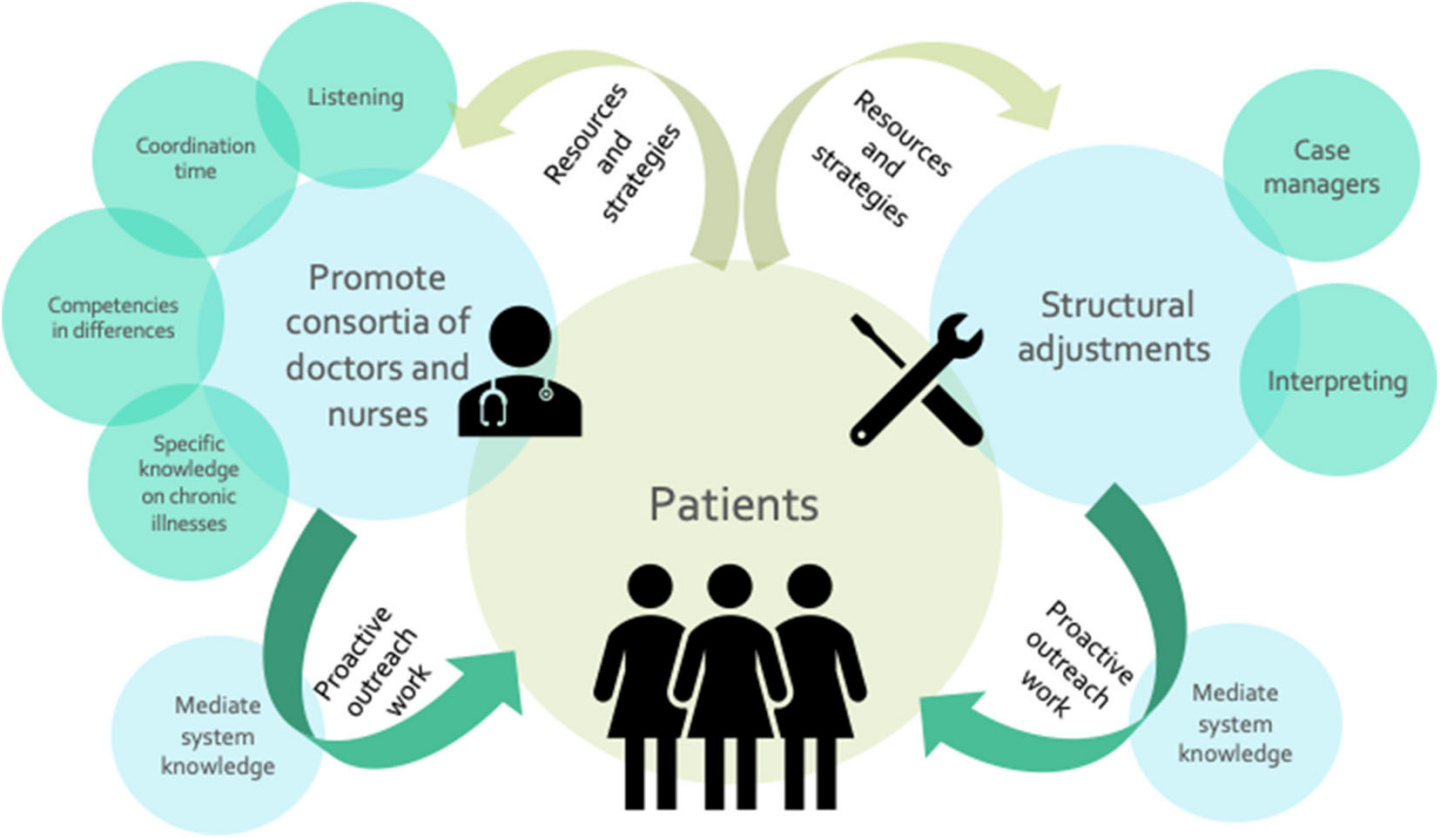
Action areas to improve competences and comprehension.

The following Figure 3 was generated as a result from the discussions during the stakeholder dialogues. It highlights factors that might contribute to improving competences and comprehension between women with chronic conditions and healthcare providers. It illustrates aspects of potentially helpful structural changes, including the need to make system and administrative knowledge accessible and to integrate different parts of care through concrete actions.

Stakeholders differentiated three action areas: structural adjustments needed, promotion of doctor-nurses-consortia, and mediation of system knowledge. Structural adjustments needed to improve comprehension refer to interpretation and translation services and the establishment of case managers. Consortia of doctors and nurses, particularly in large practices, would call for resources for coordination time, listening, and the integration of specific knowledge about chronic illness management. To increase knowledge on healthcare systems, dialogue participants called for proactive outreach work that addresses knowledge gaps early on in diagnosis and illness treatment and management.

Those aspects had been already highlighted in the interviews. Interviewees from all countries of origin admitted having little knowledge about administrative processes, rules and tasks related to healthcare or about the kind of health insurance they were paying for. Nevertheless, having acquired experience over time with local administrative procedures facilitated the participants' understanding of how the health system functions. Among the participants in our study, younger women, women whose family arrangements suggest administrative tasks are handled by other members (often their partners), or women who had more recently moved to Switzerland would still face most difficulties. They were particularly challenged in understanding health insurance models and selecting the best fitting health insurance policy among the vast array of options.

Communication was also one of the areas in which trust between patients and healthcare providers was built. Interviewees evaluated healthcare providers' communication skills based upon their ability to provide clear explanations on diagnoses and treatments, as well as their capacity to admit to their own mistakes, sincerity, and openness.

In this sense, several women from Turkey explained that they had difficulties trusting doctors in Switzerland because of the latters' tendency to point to their immigration background as a potential cause of their illness. These participants commented on how they sensed that healthcare providers treated them with less attention. Similarly, some of these women expressed the need for their expressed cultural differences to be taken into account in medical interactions. For example, some Turkish participants expressed their feelings that non-Turkish doctors did not satisfactorily understand patients from Turkey. In other words, these participants also tended to look at care interactions and differences between conventional care practices in both countries (e.g., clinical examinations) through cultural lenses and expressed the need for these differences to be understood.

The interview data had already highlighted both the beneficial and detrimental influences of relatives and acquaintances on women's access to healthcare. Some participants reported that relatives and acquaintances often played a supporting role in diverse domains, such as helping the interviewees find information, assisting with translation at doctors' offices, and taking care of insurance administrative procedures. Others lacked the time and resources to build a network that could provide them with information on the local health system, especially during their first years of residence in Switzerland.

In the Swiss healthcare system, general practitioners were perceived as key actors who serve as gatekeepers. Indeed, health policies and administrative apparatuses give them the responsibility to control patients' access to more expensive consultations with specialists. In this context, for people who need to consult general practitioners and specialists regularly, finding a general practitioner they trust is a central issue. Participants of all origins were concerned with doctors' lack of attention to the causes of their illness, doctors' refusal to conduct specific examinations, and with the perceived pressure put on general practitioners' shoulders to diminish costs and hence, reduce examination durations.

### Practice Recommendations Developed in the Stakeholder Dialogues

The stakeholder dialogues resulted in concrete practice recommendations for different areas of healthcare services. They included recommendations on (a) the reduction of barriers to access health services, such as the provision of low-threshold information services, (b) the promotion of trust between health professionals and patients, such action on communication and interaction competences, (c) improved involvement of patients and their resources in decisions on the treatment, including biographical specifics, (d) how to improve cooperation between healthcare professions, including continuous care concepts, (e) promoting the non-medical support environment, such as self-help groups and community-based approaches, and (f) improving education and training opportunities, especially for transcultural healthcare and chronic illness management.

## Discussion

MIWOCA is one of the first studies in Switzerland that used PREs to analyse healthcare services and healthcare needs among migrant women with chronic illnesses. MIWOCA manages to mirror the complexity linked to the intersectionality of the experiences of chronic illness patients and immigrants. Our focus on incorporating PREs in healthcare assessments is consistent with the World Health Organization's Framework on integrated people-centered health services, which calls for health systems to move toward prioritizing people when developing health systems (25). MIWOCA highlighted key factors and challenges related to health service use, i.e., self-perceived needs, practical experiences, (dis-)satisfaction with services, utilization patterns, and personal and social resources. MIWOCA also incorporates the views of healthcare and social service providers, who share experiences and knowledge useful for identifying system-based barriers and facilitators to healthcare utilization. As a result, the findings from our methodological approach provide new insights not only on how to understand related challenges, but also on how potentially to improve access to and quality of care in this field.

Herein, our findings echo several results from a literature review between 2005 and 2015 by Hacker et al. (34). They focused on barriers to healthcare for undocumented immigrants in various countries and found several barriers similar to the ones raised by women with a more established immigration background in our study. In the literature review, bureaucratic obstacles such as paperwork and registration systems were named as well as discriminatory practices within the health care system. At the individual level, Hacker et al. (34) also found issues of stigma and lack of social and economic capital to obtain services. They also identified recommendations to improve healthcare services, among those there are recommendations for novel insurance options, for expanding safety net services, for training service providers in better addressing immigrant populations‘needs, and for educating immigrants on navigating the system.

This study contributes to the literature by showing on the one hand the persistence of challenges in getting access to and quality of care for women with chronic illness and immigration experience and on the other hand that those challenges are perceived not only by newly arrived or undocumented immigrants, as other studies have shown (34), but also among relatively well-established groups in Switzerland, a country whose healthcare systems is among the leading ones worldwide.

## Conclusions

From a methodological perspective, MIWOCA advances research into PREs by not only including, but going beyond clinical encounters, offering data on “why” problems occur, contributing to the qualitative knowledge needed for future mixed methods research on PREs, and showing how to link data from different sources to create a foundation for participatory planning methods for improving healthcare services.

Further data analyses will be conducted to make full use of the depth of knowledge to be gained from the data. One focus will be set on applying the capability approach (11–13) to study in detail the interplay of structure and agency regarding access to and quality of healthcare services among migrant women with chronic illnesses. Other data analyses already underway will cover issues about the chronicity of illnesses, immigration facets such as countries of origin or length of stay, as well as understandings of care, and PREs. We will also use the operational Levesque model (14) to guide further development of concrete recommendations for healthcare practice.

Those more specialized findings in future analyses will come from particular disciplinary foci of the involved researchers, such as sociology, anthropology, and health systems research.

The results from MIWOCA also encompass a policy brief on access to and quality of healthcare for women with chronic conditions and immigration experience in Switzerland. The policy brief will integrate the set of recommendations on actions to be implemented. Some of these recommendations address: how to improve communication between immigrant women with chronic illness and healthcare and social service providers, how to integrate these women's resources and strategies into healthcare services for chronically ill patients, and how to strengthen the role of general practitioners and interprofessional care members in managing chronic illness and transcultural communication and interaction.

## Data Availability Statement

The raw data supporting the conclusions of this article will be made available by the authors, without undue reservation.

## Ethics Statement

The studies involving human participants were reviewed and approved by Ethics Commission of the Canton of Bern who determined no formal ethics approval was needed according to Swiss law (Basecno. 2017-00371). The patients/participants provided their written informed consent to participate in this study.

## Author Contributions

AF, SS, SC, and TA conceived and designed the study. AF, RF, TB, LD, SM, SS, and TA wrote the manuscript. AF, RF, LD, TB, SM, and SS collected the data and performed the data analyses. SC regularly provided feedback on the overall study protocol was involved in the development of the methodology, participated in the writing of the manuscript and analyzed data from the study. All authors reviewed and approved the final version of the manuscript before submission.

## Conflict of Interest

The authors declare that the research was conducted in the absence of any commercial or financial relationships that could be construed as a potential conflict of interest.
